# Physicochemical analysis of blood and urine in the course of acute kidney injury in critically ill patients: a prospective, observational study

**DOI:** 10.1186/1471-2253-13-31

**Published:** 2013-10-10

**Authors:** Alexandre Toledo Maciel, Marcelo Park, Etienne Macedo

**Affiliations:** 1 Intensive Care Unit, Department of Medical Emergencies, Hospital das Clínicas University of São Paulo, São Paulo, Brazil; 2 Department of Nephrology, Hospital das Clínicas University of São Paulo, São Paulo, Brazil; 3 Intensimed Research Group, Intensive Care Unit, Hospital São Camilo Pompéia, São Paulo, Brazil

**Keywords:** Urine biochemistry, Urine electrolytes, Physicochemical analysis, Stewart approach, Acute kidney injury, Strong ion gap, Strong ion difference, Critically ill patients

## Abstract

**Background:**

Sequential physicochemical alterations in blood and urine in the course of acute kidney injury (AKI) development have not been previously described. We aimed to describe these alterations in parallel to traditional renal and acid–base parameters.

**Methods:**

One hundred and sixty eight consecutive critically ill patients with no previous kidney disease, who had an indwelling urinary catheter at ICU admission and who remained with the catheter for at least two days without dialysis were included. A sample of blood and spot urine were collected simultaneously, once daily, until catheter removal or dialysis requirement. Traditional acid–base and renal parameters were sequentially evaluated in parallel to blood and urinary physicochemical parameters. Patients were classified during this period as having or not AKI and, for patients with AKI, duration (transient or persistent) and severity (creatinine-based AKIN stage) were evaluated.

**Results:**

One hundred and thirteen patients (67.3%) had AKI: 92 at ICU admission and 21 during the observation period. AKI development was characterized in blood by increased values of phosphate and unmeasured anions (SIG), decreased albumin, and in urine by decreased values of sodium (NaU), chloride (ClU) as well as high urinary strong ion difference (SIDu). These alterations began to occur before AKI diagnosis, and they reverted in transient AKI but remained in persistent AKI. NaU, ClU and albumin decreased, and phosphate, SIG and SIDu increased with AKI severity progression. NaU and ClU values increased again when AKIN stage 3 was reached.

**Conclusions:**

Simultaneous physicochemical analysis of blood and urine revealed standardized alterations that characterize AKI development in critically ill patients. These alterations paralleled AKI duration and severity. Future studies should consider including sequential evaluation of urine biochemistry as part of the armamentarium for AKI diagnosis and management.

## Background

Blood and urinary indices are frequently used to distinguish a transient, reversible decline in kidney function, from a persistent, structural kidney impairment (generally attributed to acute tubular necrosis (ATN)). However, discrimination based on these indices has been questioned [1-3] with no evidence of ATN in most cases [4,5]. The correlation between structural kidney damage and urinary biochemistry is also frequently questioned [1,6]. Few studies have evaluated the accuracy of urinary indices in distinguishing transient and persistent acute kidney injury (AKI), with conflicting results [7-10].

Nowadays, urinary biochemical analysis is considered of little use in the diagnosis and management of AKI, especially in sepsis [11-13]. However, preliminary results of our group showed that sequential analysis of some urinary biochemical parameters has a potential role in detect AKI even before increases in creatinine [14,15]. In addition, a physicochemical acid–base analysis of blood [16] and urine [17,18] has also been previously used to describe some of the alterations that occur when renal function is impaired. However, these studies were limited to patients with established AKI: none has focused on a sequential evaluation of these physicochemical parameters in the course of AKI, including the days preceding AKI diagnosis.

The aims of the present study were to describe the sequential analysis of blood and urinary physicochemical parameters in critically ill patients according to AKI duration (transient or persistent) and AKI severity.

## Methods

The “Hospital das Clínicas da Faculdade de Medicina da Universidade de São Paulo” ethics committee approved the study (protocol number 0093/11), and the need for informed written consent was waived by the same committee. The study was conducted prospectively in a single, mixed medical-surgical 6-bed ICU from October 2009 to November 2011. Blood laboratory exams used in this study were all collected routinely, once daily, between 8 p.m. and 10 p.m., from every patient in our ICU. These exams include arterial blood gases, arterial lactate, serum urea, creatinine (SCr), Na^+^, K^+^, Ca^2+^, Mg^2+^, Cl^-^, phosphate and albumin. In addition, starting in October 2009, we included a spot urine sample as part of the routine exams. Urine sample was only taken from patients with a urinary catheter in place when blood was being collected. We measured Na^+^ (NaU), K^+^ (KU), Cl^-^ (ClU), urea (UrU) and creatinine (CrU) levels in the spot urine sample, and a 2-h creatinine clearance was calculated using the 8 p.m. to 10 p.m. urine volume (the collection bag is emptied every 2 hours in our ICU). Urinary strong ion difference (SIDu), standard base excess (SBE), apparent strong ion difference (SIDa), effective strong ion difference (SIDe), strong ion gap (SIG), fractional excretion of sodium (FENa) and urea (FEUr) were calculated daily using the exams previously mentioned (see formulas below). For the purposes of this study, we analyzed consecutive patients who had a urinary catheter inserted before or at ICU admission and remained with the catheter for at least the first two days of routine exams in the ICU. We excluded patients who were discharged, died, had the urinary catheter removed or needed dialysis before completing these two sets of routine exams. Patients with kidney transplant, chronic renal failure, or who were readmitted to the ICU were also excluded. The observation period for each patient begins at ICU admission until indwelling urinary catheter removal (minimum of two days) or need for renal replacement therapy, which of the two that occurred first. Urinary catheter insertion and removal was at the discretion of the assistant physician and not influenced by the ongoing study.

Patients demographics, associated comorbidities, severity scores (SAPS 3 [19] and SOFA[20]), urine output and fluid balance, use of vasopressors, bicarbonate and loop diuretics, need for mechanical ventilation and renal replacement therapy during the observation period as well as ICU and hospital mortality were recorded.

### Physicochemical approach to acid–base disturbances

In the Stewart approach [21], modified by Figge [22], the difference between strong (completely dissociated) cations and anions (SID), and Atot (the total concentration of the weak acids albumin and phosphate) are the determinants of the metabolic acid–base profile. To keep electroneutrality of the solution, water molecules dissociate when there is a decrease in SID; the resulted increment in the concentration of free protons decreases pH (an acidifying effect).

In physiological conditions, the difference between strong cations and anions (SIDa) is equal to the sum of bicarbonate and the dissociated part of weak acids (SIDe). In the presence of unmeasured anions, SIDa is greater than SIDe and this difference is called strong ion gap (SIG).

The kidneys are the major regulators of plasma SID. Normally functioning kidneys are able to counteract increases in circulating acids by increasing the net acid excretion in urine. The main mechanism for urinary acid excretion is an increase in urinary ammonium (NH_4_^+^), the combination of ammonia (NH_3_), a metabolite of glutamine, and a proton (H^+^). Urinary NH_4_^+^ is usually not directly measured but inferred by the urinary strong ion difference (SIDu): NaU + KU – ClU. To keep electroneutrality, increases in urinary NH_4_^+^ are usually accompanied by increases in urinary Cl^-^ concentration, decreasing the SIDu.

We calculated standard base excess (SBE), SIDa, SIDe, SIG, SIDu, fractional excretion of sodium (FENa) and urea (FEUr) using standard formulas:

1. SBE (Van Slyke equation) (mEq/L) = 0.9287 × (HCO_3_^-^ (mmol/L) – 24.4 + 14.83 × [pH – 7.4])

2. Apparent strong ion difference (SIDa) (mEq/L) = Na^+^ (mEq/L) + K^+^ (mEq/L) + Ca^2+^ (mEq/L) + Mg^2+^ (mEq/L) – [Cl^-^ (mEq/L) + lactate^-^ (mEq/L)]

3. Effective strong ion difference (SIDe) (mEq/L) = 2.46 × 10^-8^ × PCO_2_/10^-pH^ + [(10 × albumin (g/dL)) × (0.123 × pH – 0.631)] + [(phosphate (mg/dL) × 10/30.97) × (0.309 × pH – 0.469)]

4. SIG (mEq/L) = SIDa – SIDe

5. FENa (%) = [(NaU(mEq/L)/Na^+^ (mEq/L))/(CrU (mg/dL)/SCr (mg/dL))] × 100

6. FEUr (%) = [(UrU (mg/dL)/urea (mg/dL))/(CrU (mg/dL)/SCr (mg/dL))] × 100

7. 2-h creatinine clearance (ml/min) = [(CrU (mg/dL) × (8 p.m. to 10p.m.diuresis volume (ml)))/SCr (mg/dL)]/120

### Acute kidney injury diagnosis and reversal

We defined acute kidney injury (AKI) based on the AKIN [23] creatinine criteria considering the time period during which the patient was with a urinary catheter. Baseline serum creatinine (baseSCr) was defined as the lowest SCr in the previous 3 months before ICU admission. Measured SCr was adjusted daily for cumulative fluid balance [24]. We used the lowest value between baseSCr and adjusted creatinine in the first 7 days of the study to determine if the patient had AKI at admission. To diagnose AKI during ICU stay we used the lowest SCr in the last 48 hours (reference creatinine (refSCr)). AKINmax refers to the worst AKIN stage reached during the study period.

Transient AKI was determined when SCr returned to a value lower than (SCr used for diagnosis + 0.3 mg/dL) or (SCr used for diagnosis × 1.5) within 48 hours of onset, depending on which of the two criteria made the diagnosis. Day 0 (D0) refers to the day in which AKI diagnosis was made or, for patients who did not develop AKI, the first ICU day. Days -1 (D-1) and -2 (D-2) refer respectively to 24 and 48 h before the AKI diagnosis, and days 1 (D1) and 2 (D2) refer respectively to 24 and 48 h after the AKI diagnosis or after ICU admission (for no-AKI patients). If the patient did not continue with the urinary catheter for 48 hours after AKI diagnosis, the creatinine values of those two days were retrospectively retrieved in order to define AKI reversal.

### Laboratory techniques and measurements

All samples were analyzed in the central laboratory of our institution. Serum Na^+^, K^+^, Ca^2+^ and Cl^-^ concentrations were measured using the direct ion-selective electrode technique; Mg^2+^ was measured using a colorimetric technique; phosphate was measured using an ultraviolet technique. Urea was measured with a kinetic technique both in blood and urine, and albumin was measured with a bromocresol dye colorimetric technique. NaU, KU and ClU were also measured using the direct ion-selective electrode technique, while creatinine was measured in both blood and urine using a kinetic colorimetric technique. Arterial blood gas was analyzed, and lactate was measured on the OMNI analyzer (Roche Diagnostics System, F. Hoffmann-La Roche Ltd, Basel, Switzerland).

### Statistical analyses

Continuous variables were expressed as median and 25–75 percentiles and categorical variables were expressed as absolute (n) and relative (%) frequency. Between-group differences for continuous variables were analyzed by the non-parametric Wilcoxon–Mann–Whitney test. Categorical variables were analyzed by Pearson’s χ^2^-test or Fisher’s exact test, whenever appropriate. All statistical tests were two-sided and p < 0.05 was considered significant. Statistical analyses were conducted using SPSS 19.0 (Chicago, Illinois).

## Results

### Incidence of transient and persistent AKI

**Figure 1:**
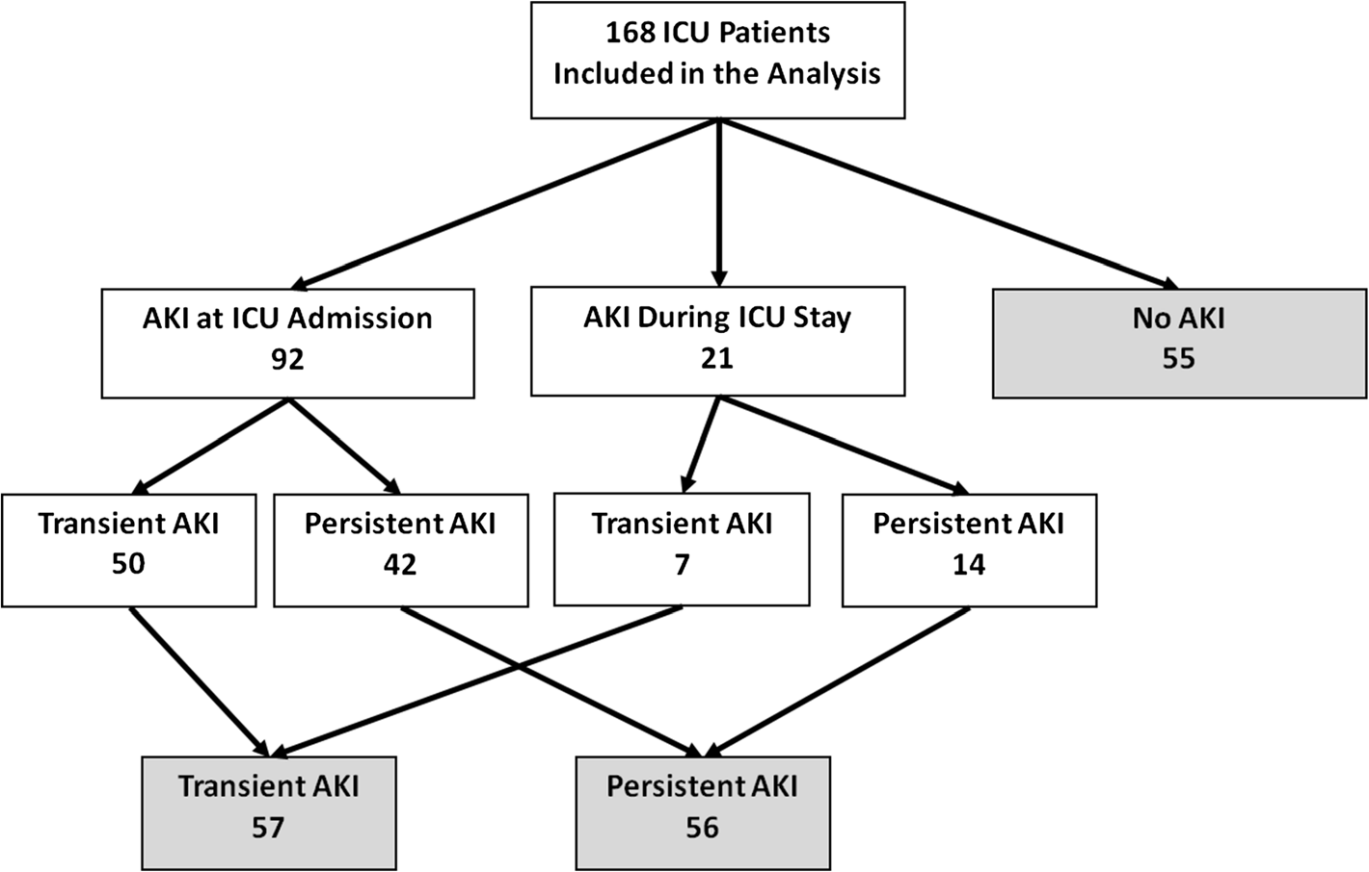
Flowchart of patients included in the analysis according to AKI duration.

A hundred and sixty eight patients were included in this analysis. During the study period 113 patients (67.3%) had AKI: 92 (81.4%) at ICU admission and 21 (18.6%) during ICU stay. Fifty out of the 92 patients (54.3%) with AKI at admission had a transient AKI and 42 (45.7%) had a persistent AKI. Seven out of 21 patients (33.3%) with AKI diagnosed during ICU stay had a transient AKI and 14 (66.7%) had a persistent AKI (Figure 1).

### Epidemiologic differences between transient and persistent AKI

General characteristics of the patients in the 3 groups are shown in Table 1. No differences were found in age, gender, ideal body weight and associated chronic organ failures between groups. Patients who developed persistent AKI had higher SAPS 3 and SOFA score at admission. BaseSCr was significantly lower in the no-AKI patients. No differences between groups were found in the percentage of patients who had used diuretics or needed mechanical ventilation. However, bicarbonate and vasopressor use was more prevalent in patients who developed persistent AKI. Only one patient in the transient AKI group required renal replacement therapy but long after complete recovery from the first transient episode of AKI. AKIN stages 2 or 3 were significantly more prevalent in persistent AKI than in transient AKI. Both ICU and hospital mortality were also significantly higher in patients with persistent AKI.

**Table 1:** Main characteristics of patients according to acute kidney injury (AKI) status during the observation period

	**No AKI (n = 55)**	**Transient AKI (n = 57)**	**Persistent AKI (n = 56)**	**P**
**Male gender**	30 (54.5%)	31 (54.4%)	32 (57.1%)	0.95
**Age (years)**	47 [29,63]	54 [38,62]	56 [45,69]	0.19
**Ideal body weight (Kg)**	56 [52,63]	54 [48,64]	55 [48,63]	0.79
**SAPS 3 (ICU admission)**	33 [27,50]	47 [37,57]	51 [33,62]	0.01
**SOFA (ICU admission)**	4 [2,7]	6 [4,8]	7 [5,10]	0.01
**Associated diseases**				
-COPD	1 (1.8%)	1 (1.8%)	1 (1.8%)	1.00
-Heart failure	4 (7.3%)	5 (8.8%)	10 (17.9%)	0.16
-Cirrhosis	3 (5.5%)	1 (1.8%)	0 (0%)	0.16
**Creatinine baseline- mg/dl**	0.63 [0.51,0.79]	0.91 [0.63,1.17]	0.84 [0.64,1.08]	<0.01
**Loop Diuretics**	17 (30.9%)	27 (47.4%)	26 (46.4%)	0.14
**Bicarbonate**	4 (7.3%)	9 (15.8%)	17 (30.4%)	0.01
**Vasopressor**	14 (25.5%)	25 (43.9%)	35 (62.5%)	<0.01
**Mechanical ventilation**	33 (60%)	35 (61.4%)	36 (64.3%)	0.89
**Renal replacement therapy**	0 (0%)	1 (1.8%)	17 (30.4%)	<0.01
**Main diagnosis**				0.01
-Severe sepsis/septic shock	8 (14.5%)	17 (29.8%)	29 (51.7%)	
-Respiratory failure	10 (18.2%)	12 (21.1%)	9 (16.0%)	
-Neurologic syndromes	9 (16.4%)	8 (14.0%)	6 (10.8%)	
-Post-operative	7 (12.7%)	7 (12.3%)	2 (3.6%)	
-Trauma	8 (14.5%)	3 (5.3%)	1 (1.8%)	
-Non-septic shock	2 (3.7%)	4 (7.0%)	2 (3.6%)	
-Others	11 (20%)	6 (10.5%)	7 (12.5%)	
**Maximum AKIN stage**				<0.01
-Stage 1		37 (64.9%)	11 (19.7%)	
-Stage 2		15 (26.3%)	25 (44.6%)	
-Stage 3		5 (8.8%)	20 (35.7%)	
**Mortality**				
-ICU	3 (5.5%)	7 (12.3%)	17 (30.4%)	0.01
-Hospital	7 (12.7%)	11 (19.3%)	25 (44.6%)	<0.01

SAPS: simplified acute physiology score; SOFA: sequential organ failure assessment; ICU: intensive care unit;

COPD: chronic obstructive pulmonary disease; AKIN: acute kidney injury network.

### Traditional renal and acid–base parameters in the course of AKI

Most AKI diagnosis were made at ICU admission, thus only a few patients had the values recorded 2 days before AKI diagnosis (n = 13). A low 2 h-creatinine clearance was already observed two days before AKI diagnosis (Table 2). Creatinine values were significantly higher in persistent AKI compared to transient AKI at the day of AKI diagnosis (D0). Transient AKI patients still had higher creatinine levels than no-AKI patients at D2. Although not statistically significant, measured 2 h-creatinine clearance was also still lower in transient AKI compared to no-AKI patients at D2. Urea levels paralleled creatinine levels on all days and within all groups. 24 h-urine output and fluid balance were similar between groups on all days. No statistical differences were found in SBE and bicarbonate levels between groups at D0, but persistent AKI patients had a slightly lower SBE at D2. Median PCO_2_ was significantly lower in persistent AKI in comparison to transient AKI at D1 and D2. Median pH levels were in normal range on all days of observation in all groups but significantly different between groups at D0 (lower in persistent AKI).

**Table 2:** Traditional renal and acid–base parameters in the course of acute kidney injury (AKI)

		**Day -2 (n = 13)**	**Day -1 (n = 21)**	**Day 0 (n = 168)**	**Day +1 (n = 164)**	**Day +2 (n = 117)**
**Urea (mg/dL)**	No-AKI	-	-	27 [21,46]	29 [20,48]	27 [19,45]
	transient	37 [34,40]	45 [40,68]	68 [51,90]	63 [42,89]	48 [34,66]
	persistent	33 [20,42]	47 [35,65]	75 [60,115]	84 [62,136]	95 [58,132]
	P			*0.00/ **0.30/ ***0.00	0.00/ 0.00/ 0.00	0.00/ 0.00/ 0.00
**Creatinine (mg/dL)**	No-AKI	-	-	0.68 [0.55,0.82]	0.69 [0.57,0.83]	0.62 [0.54,0.76]
	transient	0.66 [0.60,0.73]	0.79 [0.63,0.94]	1.24 [0.86,1.70]	0.98 [0.81,1.45]	0.82 [0.65,1.09]
	persistent	0.72 [0.58,0.80]	0.91 [0.59,1.02]	1.62 [1.17,2.55]	1.73 [1.11-2.48]	1.69 [1.07,2.38]
	P			0.00/ 0.02/ 0.00	0.00/ 0.00/ 0.00	0.00/ 0.00/ 0.02
**24 h-urine output (mL/kg/h)**	No-AKI	-	-	1.13 [0.68,1.88]	1.13 [0.81,2.24]	1.39 [0.80,1.93]
	transient	1.74 [1.40,2.15]	0.65 [0.42,1.04]	1.18 [0.79,1.95]	1.36 [0.92,2.31]	1.07 [0.68,2.08]
	persistent	1.31 [0.94,2.00]	0.87 [0.60,1.67]	0.83 [0.62,1.28]	0.97 [0.48,1.42]	0.93 [0.55,1.34]
	P			0.28/ 0.11/ 0.72	0.12/ 0.07/ 0.79	0.18/ 1.00/ 0.49
**24 h-fluid balance (mL)**	No-AKI	-	-	408 [-492,973]	220 [-419,765]	160 [-655,685]
	transient	514 [171,1130]	890 [85,968]	-119 [-770,891]	-260 [-754,455]	-64 [-1082,1190]
	persistent	841 [174,1001]	691 [30,1515]	158 [-255,922]	255 [-391,717]	27 [-697,807]
	P			0.18/0.10/ 0.08	0.10/0.05/ 0.14	0.71/0.78/ 0.76
**pH**	No-AKI	-	-	7.38 [7.33,7.42]	7.39 [7.36,7.41]	7.39 [7.37,7.42]
	transient	7.38 [7.38,7.38]	7.39 [7.31,7.43]	7.36 [7.32,7.40]	7.39 [7.35,7.41]	7.40 [7.37,7.43]
	persistent	7.42 [7.38,7.48]	7.41 [7.33,7.44]	7.35 [7.30,7.40]	7.38 [7.35,7.41]	7.39 [7.36,7.42]
	P			0.04/ 0.51/ 0.26	0.32/ 0.38/ 1.00	0.80/ 0.43/ 0.73
**PCO**_**2 **_**(mmHg)**	No-AKI	-	-	41 [34,47]	40 [35,46]	40 [35,45]
	transient	40 [38,51]	45 [35,53]	41 [33,46]	41 [35,46]	42 [37,48]
	persistent	39 [32,42]	38 [33,44]	37 [33,43]	37 [31,43]	37 [33,44]
	P			0.29/ 0.25/0.71	0.00/ 0.00/ 0.90	0.05/ 0.02/ 0.38
**SBE (mEq/L)**	No-AKI	-	-	-2.6 [-3.7,1.1]	-0.9 [-3.3,1.6]	0.2 [-2.5,2.3]
	transient	1.5 [-1.1,2.0]	-0.4 [-3.4,0.7]	-2.6 [-6.2,0.2]	-1.1 [-3.8,2.6]	1.2 [-2.1,4.6]
	persistent	0.7 [-2.7,2.6]	0.2 [-5.6,1.7]	-4.1 [-7.4,-0.4]	-3.2 [-6.6,-0.4]	-1.9 [-3.4,0.4]
	P			0.24/ 0.07/ 0.85	0.04/ 0.10/ 0.70	0.01/ 0.01/ 0.20
**Bicarbonate (mEq/l)**	No-AKI	-	-	22.4 [20.8,25.8]	23.7 [21.4,26.6]	24.5 [22.2,26.7]
	transient	26.9 [22.3,27.9]	23.4 [22.3,26.7]	22.1 [18.7,25.3]	23.6 [20.6,27.2]	25.4 [22.2,29.3]
	persistent	24.4 [21.9,26.3]	24 [19.1,26.4]	20.3 [17.5,24.5]	21.5 [17.6,24.7]	22.9 [20.5,25.3]
	P			0.07/ 0.07 / 0.85	0.027/ 0.042/ 0.847	0.00/ 0.01/ 0.20
**2 h-creatinine clearance** **(mL/min)**	No-AKI	-	-	117.8 [55.9,180.0]	111.7 [92.0,194.0]	112.7 [63.9,228.1]
	transient	57.7 [18.7-234.6]	56.9 [13.4,202.8]	66.5 [41.5,115.9]	85.2 [51.6,194.7]	76.0 [41.1,148.1]
	persistent	59.7 [32.2-114.5]	25.9 [9.0,72.5]	31.7 [19.0,49.5]	29.7 [14.1,58.3]	36.8 [15.2,69.0]
	P			0.00/ 0.00/ 0.03	0.00/ 0.00/ 0.24	0.01/ 0.35/ 0.36

Day 0: day of AKI diagnosis; SBE: standard base excess; * among the three groups; ** between transient and persistent AKI; *** between no AKI and transient AKI.

### Blood physicochemical parameters in the course of AKI

**Figure 2:**
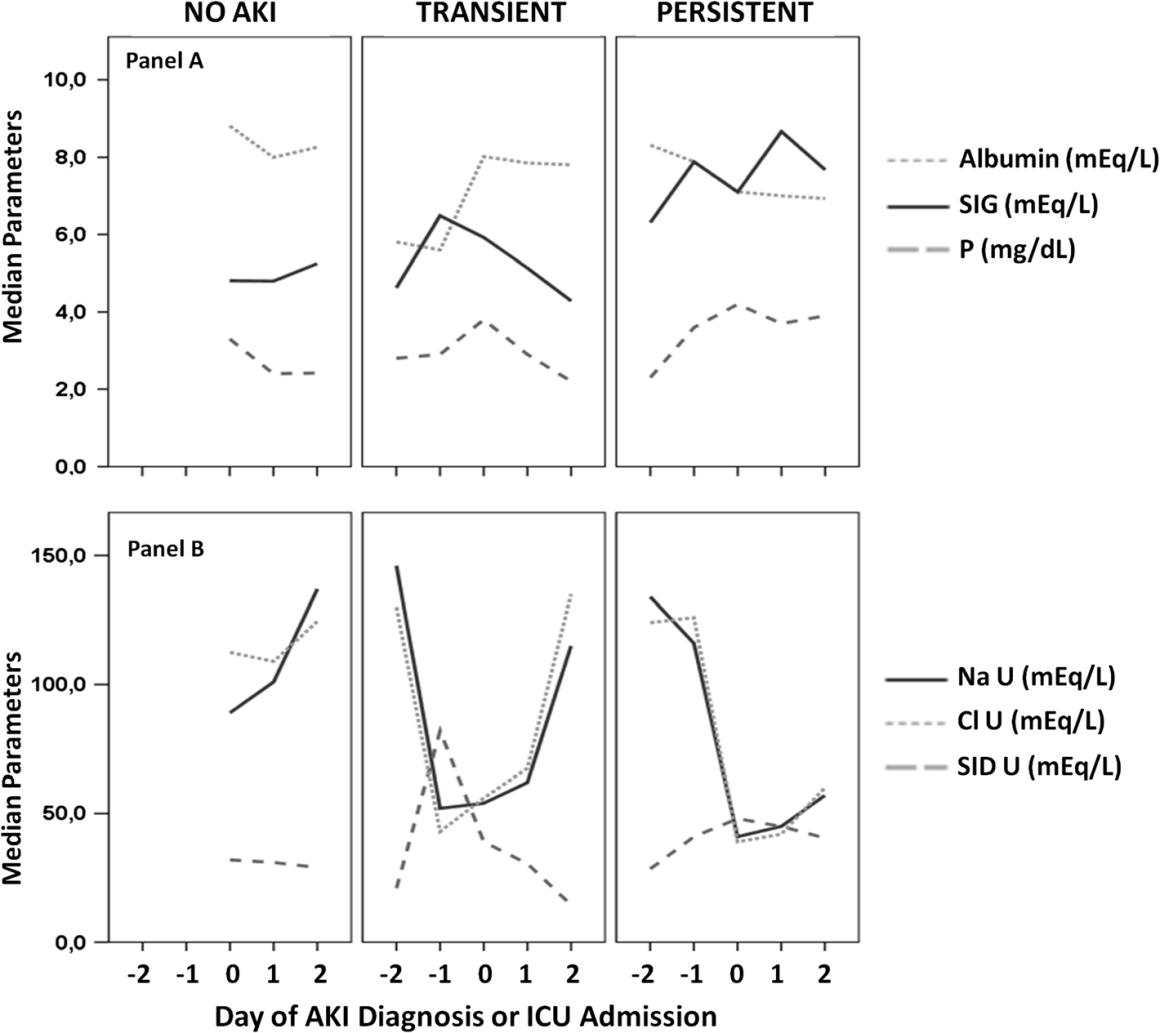
**Summary of the main blood and urinary physicochemical parameters between 2 days before (-2) until 2 days after (2) acute kidney injury (AKI) diagnosis (0), according to AKI duration.** Panel **A**. Blood parameters. Panel **B**. Urinary parameters. SIG: strong ion gap; P: phosphorus; NaU: urinary sodium; ClU: urinary chloride; SIDu: urinary strong ion difference.

Median SIDa was stable and similar in no-AKI, transient and persistent AKI patients on all days (Table 3). SIDe was lower in persistent AKI and reached significant difference between groups at D2. Both SIG and phosphate levels were higher in AKI patients at D0, decreasing to values similar to no-AKI patients at D2 in the transient group and remaining high in the persistent group (Table 3, Figure 2 – panel A). At D0, albumin values were also different between groups, the lowest values found in the persistent AKI group. At D1, albumin was significantly lower in persistent AKI in comparison to transient AKI (Table 3).

**Table 3:** Blood physicochemical parameters in the course of acute kidney injury (AKI)

		**Day -2 (n = 13)**	**Day -1 (n = 21)**	**Day 0 (n = 168)**	**Day +1 (n = 164)**	**Day +2 (n = 117)**
**SIDa (mEq/L)**	No-AKI	-	-	39.2 [35.7,42.7]	39.3 [35.9,42.9]	40.1 [37.4,43.9]
	transient	35.8 [33.7,39.8]	39.9 [37.1,41.6]	39.6 [36.0,42.7]	39.2 [36.2,43.4]	39.5 [37.0,43.3]
	persistent	39.5 [37.1,44.6]	40.2 [38.4,47.4]	38.5 [35.9,40.7]	38.8 [36.5,42.6]	39.7 [37.6,44.6]
	P			*0.75/**0.45/*** 0.63	0.96/ 0.77/ 0.92	0.89/ 0.90/ 0.90
**SIDe (mEq/L)**	No-AKI	-	-	32.9 [29.7,37.4]	33.7 [30.9,37.4]	34.6 [32.1,38.3]
	transient	35.5 [31.1,36.9]	32.2 [30.2,34.7]	33.0 [28.3,36.3]	34.7 [30.6,37.2]	35.9 [30.9,40.0]
	persistent	34.1 [31.8,37.1]	33.8 [29.3,38.2]	29.8 [27.4,35.0]	31.0 [26.8,34.7]	32.4 [29.7,35.3]
	P			0.07/ 0.07/ 0.85	0.12/ 0.04/ 0.38	0.03/ 0.03/ 0.73
**SIG (mEq/L)**	No-AKI	-	-	4.8 [2.9,7.3]	4.8 [3.3,6.4]	5.2 [4.1,7.1]
	transient	4.6 [3.0,4,9]	6.5 [5.7,8.2]	5.9 [4.1,7.3]	5.1 [3.5,7.1]	4.3 [2.0,6.6]
	persistent	6.3 [4.0,7.7]	7.9 [5.0,9.1]	7.1 [4.7,9.7]	8.7 [5.8,10.8]	7.6 [5.2,10.3]
	P			0.01/ 0.26/ 0.22	0.00/ 0.00/ 0.33	0.01/ 0.00/ 0.12
**Phosphate**^**– **^**(mEq/L)**	No-AKI	-	-	1.9 [1.3,2.3]	1.4 [1.2,1.9]	1.4 [1.1,1.9]
	transient	1.6 [1.5,2.0]	1.7 [1.4,2.0]	2.2 [1.6,2.8]	1.7 [1.3,2.0]	1.3 [1.1,2.0]
	persistent	1.4 [1.3,1.5]	2.1 [1.3,2.5]	2.4 [1.7,3.1]	2.2 [1.6,2.8]	2.2 [1.6,2.7]
	P			0.02/ 0.22/ 0.06	0.00/ 0.01/ 0.12	0.00/ 0.00/ 0.55
**Albumin**^**– **^**(mEq/l)**	No-AKI	-	-	8.8 [6.8,9.8]	8.0 [6.6,9.3]	8.3 [6.9,9.4]
	transient	5.8 [5.3,6.4]	5.6 [5.2,6.4]	8.0 [7.1,9.0]	7.9 [6.8,9.2]	7.8 [6.9,9.0]
	persistent	8.3 [7.5,8.9]	7.9 [7.1,8.8]	7.1 [6.2,8.3]	7.0 [6.2,7.9]	7.0 [6.5,8.1]
	P			0.01/0.07/ 0.19	0.00/ 0.02/ 0.50	0.14/ 0.22/ 0.55

Day 0: day of AKI diagnosis; SIDa: apparent strong ion difference; SIDe: effective strong ion difference; SIG: strong ion gap (=SIDa-SIDe).

* among the three groups; ** between transient and persistent AKI; *** between no-AKI and transient AKI.

### Urinary physicochemical parameters in the course of AKI

#### ***Spot urinary electrolytes***

NaU, KU, ClU, NaU/KU ratio and SIDu were also evaluated from D-2 to D2 (Table 4). At D0, both NaU and ClU were significantly different between groups, with the lowest values in persistent AKI (Table 4, Figure 2 – panel B). This difference between groups remained until D2. NaU/KU ratio was also different between groups reaching statistical significance at D1 and D2 (lowest values in persistent AKI). AKI development was characterized in both AKI groups by decreases in NaU and ClU values as well as in NaU/KU ratio on the 2 days preceding AKI diagnosis. In transient AKI, decreases in NaU, ClU and NaU/KU ratio were also transient (Table 4, Figure 2- panel B); in persistent AKI, their values remained low. This pattern was not altered by diuretic use during the observation period (Additional file 1: Figure S1). No differences were found in the KU values between and within groups. SIDu had a transitory increase in transient AKI but a sustained increase in persistent AKI. At D2, patients with persistent AKI had a significantly higher SIDu than patients with a transient AKI (Table 4, Figure 2- panel B).

**Table 4:** Urinary physicochemical parameters in the course of acute kidney injury (AKI)

		**Day -2 (n = 13)**	**Day -1 (n = 21)**	**Day 0 (n = 168)**	**Day +1 (n = 164)**	**Day +2 (n = 117)**
**NaU (mEq/L)**	No-AKI	-	-	89 [44,167]	101 [51,143]	137 [95,195]
	transient	146 [79,175]	52 [31,127]	54 [22,90]	62 [30,123]	115 [72,180]
	persistent	134 [39,175]	116 [48,164]	41 [18,84]	45 [22,88]	58 [23,109]
	P			*0.00/ **0.09/ ***0.00	0.06/ 0.44/ 0.15	0.00/ 0.01/ 0.55
**ClU (mEq/L)**	No-AKI	-	-	113 [39,173]	109 [52,163]	125 [95,184]
	transient	130 [113,187]	43 [39,170]	56 [23,101]	68 [31,135]	135 [80,191]
	persistent	124 [53,177]	126 [47,155]	39 [17,84]	42 [15,97]	60 [22,103]
	P			0.00/ 0.12/ 0.00	0.00/ 0.21/ 0.07	0.00/ 0.00/ 0.48
**KU (mEq/L)**	No-AKI	-	-	45 [31,70]	40 [29,53]	33 [23,46]
	transient	49 [39,52]	52 [17,88]	36 [25,59]	38 [26,55]	36 [26,48]
	persistent	37 [20,54]	41 [30,63]	48 [29,65]	42 [30,64]	37 [24,64]
	P			0.54/ 0.34/ 0.34	0.61/ 0.34/ 0.50	0.51/ 0.55/ 0.43
**NaU/KU ratio**	No-AKI	-	-	2.0 [0.7,3.8]	2.6 [0.7,4.2]	4.6 [2.1,7.9]
	transient	2.9 [2.8,3.0]	1.2 [0.5,5.5]	1.2 [0.5,2.8]	1.4 [0.5,3.7]	4.2 [1.8,6.0]
	persistent	4.5 [1.5,6.0]	2.1 [1.2,4.9]	0.8 [0.4,2.6]	1.2 [0.3,2.7]	1.4 [0.4,3.2]
	P			0.17/ 0.34/ 0.18	0.01/ 0.44/ 0.07	0.00/ 0.09/ 0.55
**SIDu (mEq/L)**	No-AKI	-	-	32 [15,88]	31 [-9,63]	29 [-2,67]
	transient	21 [-5,71]	82 [9,107]	39 [16,58]	31 [1,57]	15 [-9,47]
	persistent	29 [7,64]	41 [31,71]	48 [26,67]	45 [29,68]	39 [28,64]
	P			0.21/ 0.44/ 0.44	0.16/ 0.12/ 0.92	0.00/ 0.00/ 0.22
**FENa (%)**	No-AKI	-	-	0.5 [0.2,1.3]	0.5 [0.2,0.9]	1.0 [0.3,2.0]
	transient	0.6 [0.3,2.0]	0.5 [0.3,0.6]	0.6 [0.2,1.3]	0.5 [0.2,1.2]	1.0 [0.3,1.7]
	persistent	1.2 [0.3,4.8]	1.5 [0.4,2.4]	0.7 [0.2,1.7]	0.9 [0.2,2.3]	0.7 [0.3,3.5]
	P			0.16/ 0.34/ 0.57	0.34/ 0.25/ 0.92	0.47/ 0.43/ 0.81
**FEUr (%)**	No-AKI	-	-	32.1 [23.0,42.4]	35.7 [25.8,43.4]	35.8 [27.1,44.3]
	transient	38.6 [17.4,49.2]	23.1 [19.7,40.0]	26.5 [16.7,42.6]	34.1 [26.2,44.0]	36.2 [26.9,49.8]
	persistent	40.2 [30.6,48.8]	23.9 [21.2,43.2]	29.2 [19.8,36.8]	33.3 [21.8,43.6]	32.4 [24.9,43.8]
	P			0.25/ 0.56/ 0.21	0.77/ 0.92/ 0.70	0.11/ 0.10/ 0.81

Day 0: day of AKI diagnosis; NaU: spot urinary sodium; ClU: spot urinary chloride; KU: spot urinary potassium; SIDu: spot urinary strong ion difference;

FENa: fractional excretion of sodium; FEUr: fractional excretion of urea.

* among the three groups; ** between transient and persistent AKI; *** between no-AKI and transient AKI.

#### ***Fractional excretion of electrolytes***

No significant differences were found in FENa and FEUr between or within groups on all days (Table 4). This pattern was similar in patients who had and had not used diuretics in the observation period (Additional file 2: Figure S2).

### Blood and urinary physicochemical parameters and AKI severity

**Figure 3:**
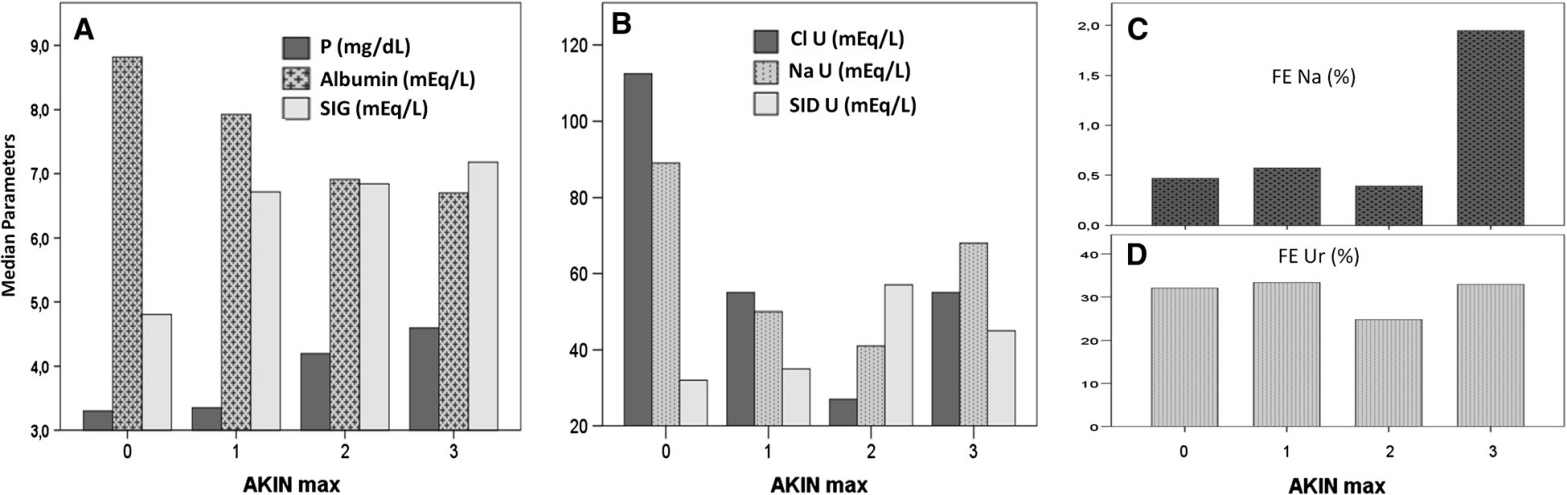
**Blood and urinary physicochemical parameters at the day of maximum AKIN stage of acute kidney injury (AKI) (AKINmax).** Panel **A**. Blood parameters: Phosphorus (P), Albumin and Strong ion gap (SIG). Panel **B**. Urinary sodium (NaU), chloride (ClU) and strong ion difference (SIDu) Panel **C**. Fractional excretion of sodium (FENa) Panel **D**. Fractional excretion of urea (FEUr). AKIN: Acute Kidney Injury Network.

To evaluate if variations in blood and urinary physicochemical parameters were proportional to the degree of AKI severity, we compared these parameters among patients who reached AKINmax stage 1, 2 or 3 during the observation period (Figure 3). We compared the values of these parameters on the day that AKINmax was reached for each patient; patients with no-AKI served as controls, with ICU values at admission used for comparison. Of 113 patients with AKI, 48 (42.5%) had an AKINmax stage 1, 40 (35.4%) stage 2 and 25 (22.1%) stage 3 (Table 1). Both SIG and phosphate increased, and albumin decreased with AKI severity progression (Figure 3A). Both NaU and ClU values decreased with progression of AKI severity except when AKINmax stage 3 was reached (Figure 3B). SIDu increased until stage 2 but decreased in stage 3 (Figure 3B). Median values of FENa were low (< 1%) in no-AKI and AKINmax stages 1 and 2 but high values were observed in AKINmax stage 3 (Figure 3C). Median FEUr values had only slight variations across AKINmax stages (Figure 3D). Median urine output (ml/kg/h) was not different across AKINmax stages (1.13, 1.12, 0.97, 0.99 respectively).

## Discussion

Urinary and blood indices have long been used as tools to distinguish functional and structural AKI [25,26]. However, the relevance of these indices is questioned by many, mainly because their values are widely variable, influenced by medications (diuretics [8], aminoglycosides [27], contrast [28]), concomitant diseases (cirrhosis [29], sepsis [30], rabdomiolysis [31]) and with a significant dissociation from renal histopathological findings [6]. However, most previous studies in this area were small and with a non-sequential evaluation of these parameters [11]. In our study, we demonstrated that there is a standard behavior of some parameters during AKI development: decreases in NaU and ClU, and increases in SIG, phosphate and SIDu. Since AKI duration is relevant to prognosis (a finding confirmed by our results), we have also demonstrated that transient AKI (creatinine elevation for less than 48 hours) is followed by equally transient alterations in these parameters both in blood and urine (Tables 3 and 4, Figure 2). Persistent AKI, on the other hand, was characterized by persistent alterations in these parameters or, at least, by a slower recovery.

Increases in SIG and phosphate are the main sources of metabolic acidosis in AKI, usually counterbalanced by hypoalbuminemia [16]. Notably, in our study, SIG and phosphate levels were significantly but only modestly increased in AKI patients in comparison to no-AKI patients (Table 3). It is noteworthy that no-AKI patients had an increased SIG (around 5 mEq/L) probably related to critical illness itself. In AKI patients, additional modest increases in SIG (2–3 mEq/L) and phosphate (0.5-1 mEq/L) may be signs of incipient renal dysfunction. In addition, the majority of our AKI patients were not acidemic nor had very low levels of SBE. Possible explanations for this were (a) low levels of albumin in AKI patients, counterbalancing the increased acid load of SIG and phosphate, and (b) greater bicarbonate use in AKI patients, although it was used in the minority of the patients (Table 1), even in persistent AKI. Hence, metabolic acidosis with acidemia is expected to be present only in advanced AKI or in the presence of other concomitant sources of metabolic acidosis, such as tissue hypoperfusion, which is likely to be present specially in persistent AKI due to the high prevalence of vasopressor use in this group.

We have also shown that, in parallel to the blood acid–base and physicochemical alterations that follow AKI development, some relevant alterations also occur in urine composition (Table 4), even in the presence of acceptable urine outputs (Table 2). Since some of these urinary alterations (decreases in NaU, ClU and NaU/KU ratio, and increases in SIDu) seem to precede increases in creatinine, they may aid in early AKI diagnosis. Decreases in NaU and ClU were previously considered part of so-called “pre-renal” AKI. Spot NaU values lower than 20 mEq/L were classically considered one of the major markers of functional AKI [25]; values above 40 mEq/L were indicative of structural AKI impairment mainly due to ATN. This paradigm, however, has been criticized by many authors [1,2,32], especially in sepsis [2]. Our data suggest that progressive decreases in NaU and ClU values may be viewed as part of AKI development in critically ill patients. Increases in AKI severity were also accompanied by more significant decreases in NaU and ClU values. The exceptions were patients who have reached AKIN stage 3 (Figure 3), in which increased values of NaU and FENa might suggest a global tubular dysfunction not present in less severe cases of AKI. In AKIN stages 1 and 2, urinary alterations were more compatible with a predominantly glomerular impairment in the presence of preserved tubular function (at least in the distal tubules). These findings do not imply that there was no tubular injury in less severe forms of AKI. Tubular injury as an early event in AKI has been demonstrated previously by many studies using blood and urinary biomarkers, even in those AKI previously considered “pre-renal” [33,34]. However, studies using urinary microscopy [35,36] or biomarkers [33] simultaneously with urinary biochemistry (to demonstrate tubular injury) have found FENa < 1% in the majority of the patients, suggesting a dissociation between tubular injury and function. Low NaU/KU ratios and low FENa (Table 4) may be signs of preserved mechanisms of renal adaptation to hemodynamic insults, in which avid Na^+^ retention is followed by K^+^ secretion in the distal tubules. In fact, neurohormonal mechanisms, including renin-angiotensin-aldosterone system activation, seem to be major characteristics of AKI [37]. Nejat et al. [33] speculated that tubular injury is a heterogeneous process in that residual normally functioning nephrons may mask or compensate for others already disabled. Our results suggest that global tubular function is only clearly compromised in very severe and advanced AKI stages, in which oliguria or very significant elevations in SCr levels make the AKI diagnosis easy but late. This “U” shape behavior of NaU in AKI progression, with higher values both in no-AKI and in AKIN stage 3, is one possible reason why isolated values of NaU are difficult to interpret. However, a very low NaU value should prompt an evaluation of factors that may stress kidney function. In addition, we observed that very high NaU values (above its normal plasma range) were rarely found in AKI patients in our study (Table 4). Median urine output was similar among different AKINmax stages; it is possible that diuretics contributed to this similar urine volume at the day that different AKINmax stages were reached. This finding highlights the need to access not only urine volume but sodium and chloride content in the urine.

FEUr has been suggested as a more precise parameter during diuretic use [7] and useful as a diagnostic index in AKI [9]. Its value, however, was similar between groups in our study, even when only patients who have used diuretics were evaluated (Additional file 2: Figure S2). This result is in agreement with two previous studies that also did not find utility in FEUr to discern transient and persistent AKI [8,10]. High SIDu values may suggest an inability of the injured kidneys to properly acidify urine (less ammonium excretion), and this was characteristic of AKI in our study (Table 4, Figure 3B). Previous studies [17,18] also suggest that SIDu measurement may alert for the presence of an impaired renal function in the presence of metabolic acidosis. Negative SIDu values, although infrequent, seem to be restricted to patients without AKI or with resolved AKI in our study (Table 4).

The high incidence of AKI in our study (67.3%), especially at admission, highlights the problem in considering admission creatinine as the baseSCr: many cases of AKI are potentially not recognized. A similar high AKI percentage was also found in a recent multicenter study in predominantly medical ICUs [10]. Interestingly, baseSCr was significantly lower in no-AKI patients, a finding also present in a study by Schinstock et al. [36]. This was not the focus of our study, but it could be that patients who developed AKI had lower basal glomerular filtration rate, albeit still in the normal range.

Our study has some limitations. It was completed in a single center, with a heterogeneous population of critically ill patients, so the reason for AKI development may be quite variable, and imprecision is expected regarding the moment that the kidneys were first injured. Besides that, only patients with an indwelling urinary catheter at admission were included which can introduce a bias in the analysis. We believe that we have selected the most critically ill patients and, therefore, the patients more likely to develop AKI during ICU stay. On the other hand, a heterogeneous population increases the chance that our results can also be found in other general ICUs. We decided to use only creatinine-based AKIN criteria which certainly excluded some AKI diagnosis due only to oliguria. Although a limitation, this increases the chance that our results may be useful for patients without an indwelling urinary catheter, such as patients in the wards, where urinary flow is usually not available. The high percentage of AKI diagnosis at admission prevented us from better exploring the possible role of some of the parameters studied in early AKI diagnosis since only a few patients had data of D-2 and D-1 available for analysis. Diuretics are always a confounding factor in urinary biochemistry, but we demonstrated similar results in patients that have or have not used diuretics during the observation period (Additional file 1: Figures S1 and Additional file 2: Figure S2). In addition, lower NaU values in the early phase of AKI could not be attributed to diuretic use since diuretics usually increase NaU values, both in transient and persistent AKI [8]. A low NaU value, even with diuretic use, is probably a marker of AKI development.

## Conclusions

The early phase of AKI development in critically ill patients is usually characterized by decreased values of NaU, ClU and serum albumin as well as increased values of SIG, phosphate and SIDu. Some of these alterations may begin to occur earlier than creatinine elevation. Transient and persistent AKI have similar alterations in these blood and urinary physicochemical parameters, differing only in their duration and severity. The degree of alterations in these parameters is also associated with the AKIN stage progression. Hence, sequential physicochemical analysis of blood and urine should be better explored in future studies in order to define the utility of these parameters as additional tools in AKI diagnosis and management.

## Competing interest

The authors declare that they have no conflicts of interests.

## Authors’ contributions

ATM conceived the study idea and proposal, developed the study design, collected the data and wrote the initial manuscript. MP collected the data and performed the data analysis. EM conceived the study idea and proposal, performed the data analysis and wrote the manuscript. All authors agreed to the final version of the manuscript and agreed to submit it for publication.

## Pre-publication history

The pre-publication history for this paper can be accessed here:

http://www.biomedcentral.com/1471-2253/13/31/prepub

## Supplementary Material

Additional file 1: Figure S1 Urinary sodium (NaU) between 2 days before (-2) until 2 days after (2) acute kidney injury (AKI) diagnosis (0), according to AKI duration and diuretic use during the observation period. Click here for file

Additional file 2: Figure S2 Fractional excretion of urea (FEUr) between 1 day before (-1) until 2 days after (2) acute kidney injury (AKI) diagnosis (0), according to AKI duration and diuretic use during the observation period. Click here for file

## References

[B1] Kellum JA Prerenal azotemia: still a useful concept? Crit Care Med 2007 35 1630 1631 10.1097/01.CCM.0000266794.57111.01 17522545

[B2] Bellomo R Bagshaw S Langenberg C Ronco C Pre-renal azotemia: a flawed paradigm in critically ill septic patients? Contrib Nephrol 2007 156 1 9 17464109 10.1159/000102008

[B3] Rachoin JS Daher R Moussallem C Milcarek B Hunter K The fallacy of the BUN:creatinine ratio in critically ill patients Nephrol Dial Transplant 2012 27 2248 2254 10.1093/ndt/gfr705 22207331

[B4] Rosen S Heyman SN Difficulties in understanding human "acute tubular necrosis": limited data and flawed animal models Kidney Int 2001 60 1220 1224 10.1046/j.1523-1755.2001.00930.x 11576335

[B5] Langenberg C Bagshaw SM May CN Bellomo R The histopathology of septic acute kidney injury: a systematic review Crit Care 2008 12 R38 10.1186/cc6823 18325092 PMC2447560

[B6] Bagshaw SM Langenberg C Wan L May CN Bellomo R A systematic review of urinary findings in experimental septic acute renal failure Crit Care Med 2007 35 1592 1598 10.1097/01.CCM.0000266684.17500.2F 17452939

[B7] Carvounis CP Nisar S Guro-Razuman S Significance of the fractional excretion of urea in the differential diagnosis of acute renal failure Kidney Int 2002 62 2223 2229 10.1046/j.1523-1755.2002.00683.x 12427149

[B8] Pepin MN Bouchard J Legault L Ethier J Diagnostic performance of fractional excretion of urea and fractional excretion of sodium in the evaluations of patients with acute kidney injury with or without diuretic treatment Am J Kidney Dis 2007 50 566 573 10.1053/j.ajkd.2007.07.001 17900456

[B9] Dewitte A Biais M Petit L Cochard JF Hilbert G Fractional excretion of urea as a diagnostic index in acute kidney injury in intensive care patients J Crit Care 2012 27 205 210 10.1016/j.jcrc.2012.02.018 22520491

[B10] Darmon M Vincent F Dellamonica J Schortgen F Gonzalez F Diagnostic performance of fractional excretion of urea in the evaluation of critically ill patients with acute kidney injury: a multicenter cohort study Crit Care 2011 15 R178 10.1186/cc10327 21794161 PMC3387621

[B11] Bagshaw SM Langenberg C Bellomo R Urinary biochemistry and microscopy in septic acute renal failure: a systematic review Am J Kidney Dis 2006 48 695 705 10.1053/j.ajkd.2006.07.017 17059988

[B12] Bellomo R Kellum JA Ronco C Acute kidney injury Lancet 2012 380 756 766 10.1016/S0140-6736(11)61454-2 22617274

[B13] Prowle J Bagshaw SM Bellomo R Renal blood flow, fractional excretion of sodium and acute kidney injury: time for a new paradigm? Curr Opin Crit Care 2012 18 585 592 10.1097/MCC.0b013e328358d480 22954663

[B14] Maciel AT Park M Early diagnosis of acute kidney injury in a critically ill patient using a combination of blood and urinary physicochemical parameters Clinics (Sao Paulo) 2012 67 525 526 10.6061/clinics/2012(05)21 22666801 PMC3351253

[B15] Maciel AT Park M Macedo E Urinary electrolyte monitoring in critically ill patients: a preliminary, observational study Rev Bras Ter Intensiva 2012 24 236 246 10.1590/S0103-507X2012000300006 23917824

[B16] Rocktaeschel J Morimatsu H Uchino S Goldsmith D Poustie S Acid–base status of critically ill patients with acute renal failure: analysis based on Stewart-Figge methodology Crit Care 2003 7 R60 10.1186/cc2333 12930557 PMC270700

[B17] Masevicius FD Tuhay G Pein MC Ventrice E Dubin A Alterations in urinary strong ion difference in critically ill patients with metabolic acidosis: a prospective observational study Crit Care Resusc 2010 12 248 254 21143085

[B18] Moviat M Terpstra AM van der Hoeven JG Pickkers P Impaired renal function is associated with greater urinary strong ion differences in critically ill patients with metabolic acidosis J Crit Care 2011 27 160 255 10.1016/j.jcrc.2011.05.028 21798700

[B19] Moreno RP Metnitz PG Almeida E Jordan B Bauer P SAPS 3–From evaluation of the patient to evaluation of the intensive care unit. Part 2: Development of a prognostic model for hospital mortality at ICU admission Intensive Care Med 2005 31 1345 1355 10.1007/s00134-005-2763-5 16132892 PMC1315315

[B20] Vincent JL Moreno R Takala J Willatts S De Mendonca A The SOFA (Sepsis-related Organ Failure Assessment) score to describe organ dysfunction/failure. On behalf of the Working Group on Sepsis-Related Problems of the European Society of Intensive Care Medicine Intensive Care Med 1996 22 707 710 10.1007/BF01709751 8844239

[B21] Stewart PA Modern quantitative acid–base chemistry Can J Physiol Pharmacol 1983 61 1444 1461 10.1139/y83-207 6423247

[B22] Figge J Rossing TH Fencl V The role of serum proteins in acid–base equilibria J Lab Clin Med 1991 117 453 467 2045713

[B23] Mehta RL Kellum JA Shah SV Molitoris BA Ronco C Acute Kidney Injury Network: report of an initiative to improve outcomes in acute kidney injury Crit Care 2007 11 R31 10.1186/cc5713 17331245 PMC2206446

[B24] Macedo E Bouchard J Soroko SH Chertow GM Himmelfarb J Fluid accumulation, recognition and staging of acute kidney injury in critically-ill patients Crit Care 2010 14 R82 10.1186/cc9004 20459609 PMC2911707

[B25] Schrier RW Wang W Poole B Mitra A Acute renal failure: definitions, diagnosis, pathogenesis, and therapy J Clin Invest 2004 114 5 14 15232604 10.1172/JCI22353 PMC437979

[B26] Schrier RW Diagnostic value of urinary sodium, chloride, urea, and flow J Am Soc Nephrol 2011 22 1610 1613 10.1681/ASN.2010121289 21852582 PMC3171932

[B27] Diskin CJ Stokes TJ Dansby LM Radcliff L Carter TB Toward the optimal clinical use of the fraction excretion of solutes in oliguric azotemia Ren Fail 2010 32 1245 1254 10.3109/0886022X.2010.517353 20954990

[B28] Fang LS Sirota RA Ebert TH Lichtenstein NS Low fractional excretion of sodium with contrast media-induced acute renal failure Arch Intern Med 1980 140 531 533 10.1001/archinte.1980.00330160091033 7362385

[B29] Arroyo V Fernandez J Gines P Pathogenesis and treatment of hepatorenal syndrome Semin Liver Dis 2008 28 81 95 10.1055/s-2008-1040323 18293279

[B30] Vaz AJ Low fractional excretion of urine sodium in acute renal failure due to sepsis Arch Intern Med 1983 143 738 739 10.1001/archinte.1983.00350040128017 6838295

[B31] Corwin HL Schreiber MJ Fang LS Low fractional excretion of sodium. Occurrence with hemoglobinuric- and myoglobinuric-induced acute renal failure Arch Intern Med 1984 144 981 982 10.1001/archinte.1984.00350170131022 6712414

[B32] Maciel AT Breaking old and new paradigms regarding urinary sodium in acute kidney injury diagnosis and management Crit Care 2013 17 115 10.1186/cc11926 23384365 PMC4056518

[B33] Nejat M Pickering JW Devarajan P Bonventre JV Edelstein CL Some biomarkers of acute kidney injury are increased in pre-renal acute injury Kidney Int 2012 81 1254 1262 10.1038/ki.2012.23 22418979 PMC3365288

[B34] Doi K Katagiri D Negishi K Hasegawa S Hamasaki Y Mild elevation of urinary biomarkers in prerenal acute kidney injury Kidney Int 2012 82 1114 1120 10.1038/ki.2012.266 22854644

[B35] Bagshaw SM Haase M Haase-Fielitz A Bennett M Devarajan P A prospective evaluation of urine microscopy in septic and non-septic acute kidney injury Nephrol Dial Transplant 2012 27 582 588 10.1093/ndt/gfr331 21669886

[B36] Schinstock CA Semret MH Wagner SJ Borland TM Bryant SC Urinalysis is more specific and urinary neutrophil gelatinase-associated lipocalin is more sensitive for early detection of acute kidney injury Nephrol Dial Transplant 2013 28 1175 1185 10.1093/ndt/gfs127 22529161

[B37] Wen X Murugan R Peng Z Kellum JA Pathophysiology of acute kidney injury: a new perspective Contrib Nephrol 2010 165 39 45 20427954 10.1159/000313743

