# Supplementary figures and images for: Physicochemical analysis of blood and urine in the course of acute kidney injury in critically ill patients: a prospective, observational study
Source: BMC Anesthesiol. 2013 Oct 10;13:31. doi: 10.1186/1471-2253-13-31 (PMC3851869; doi:10.1186/1471-2253-13-31)

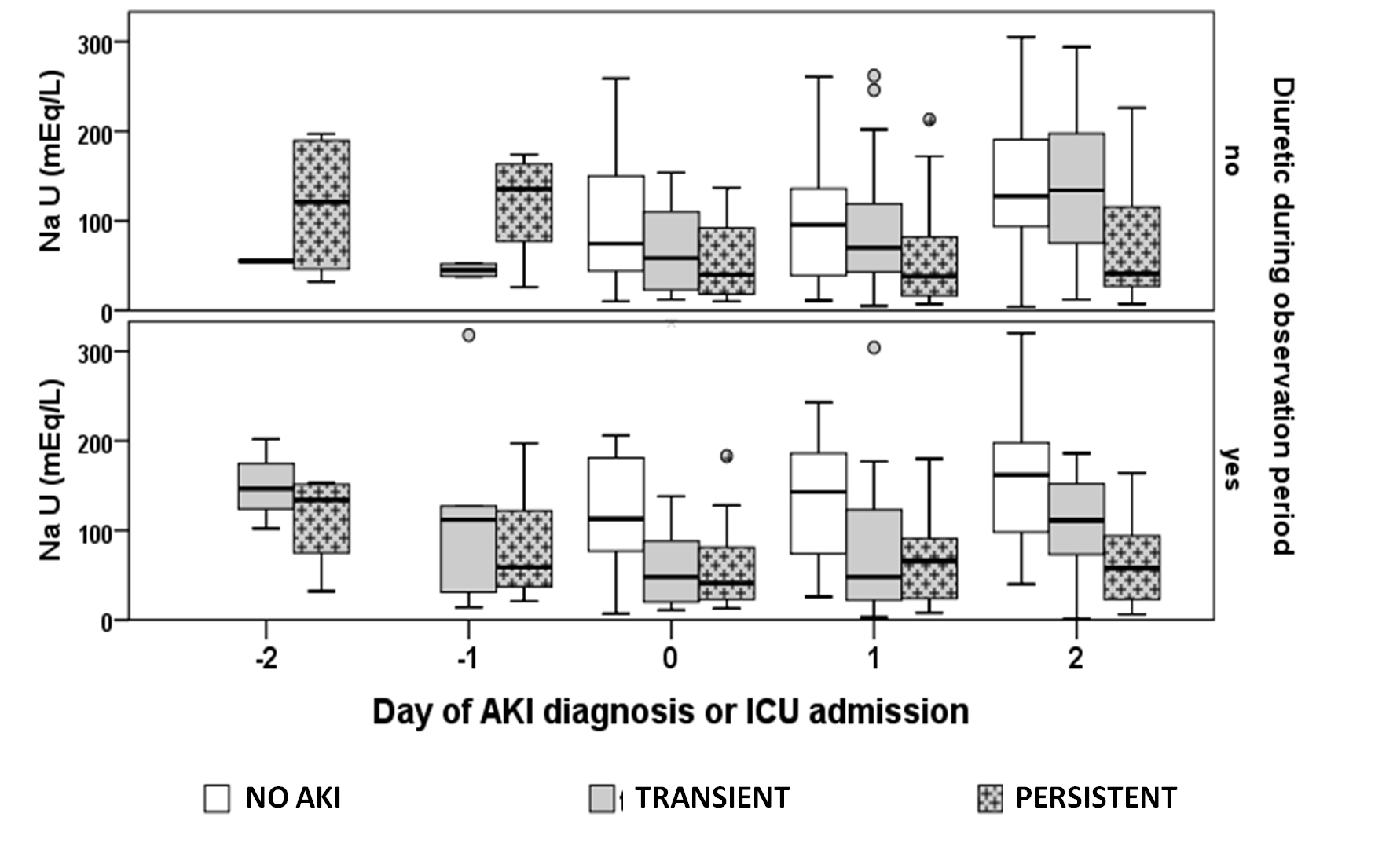

Supplement: Additional file 1: Figure S1 — Urinary sodium (NaU) between 2 days before (-2) until 2 days after (2) acute kidney injury (AKI) diagnosis (0), according to AKI duration and diuretic use during the observation period. [file 1471-2253-13-31-S1.tiff]

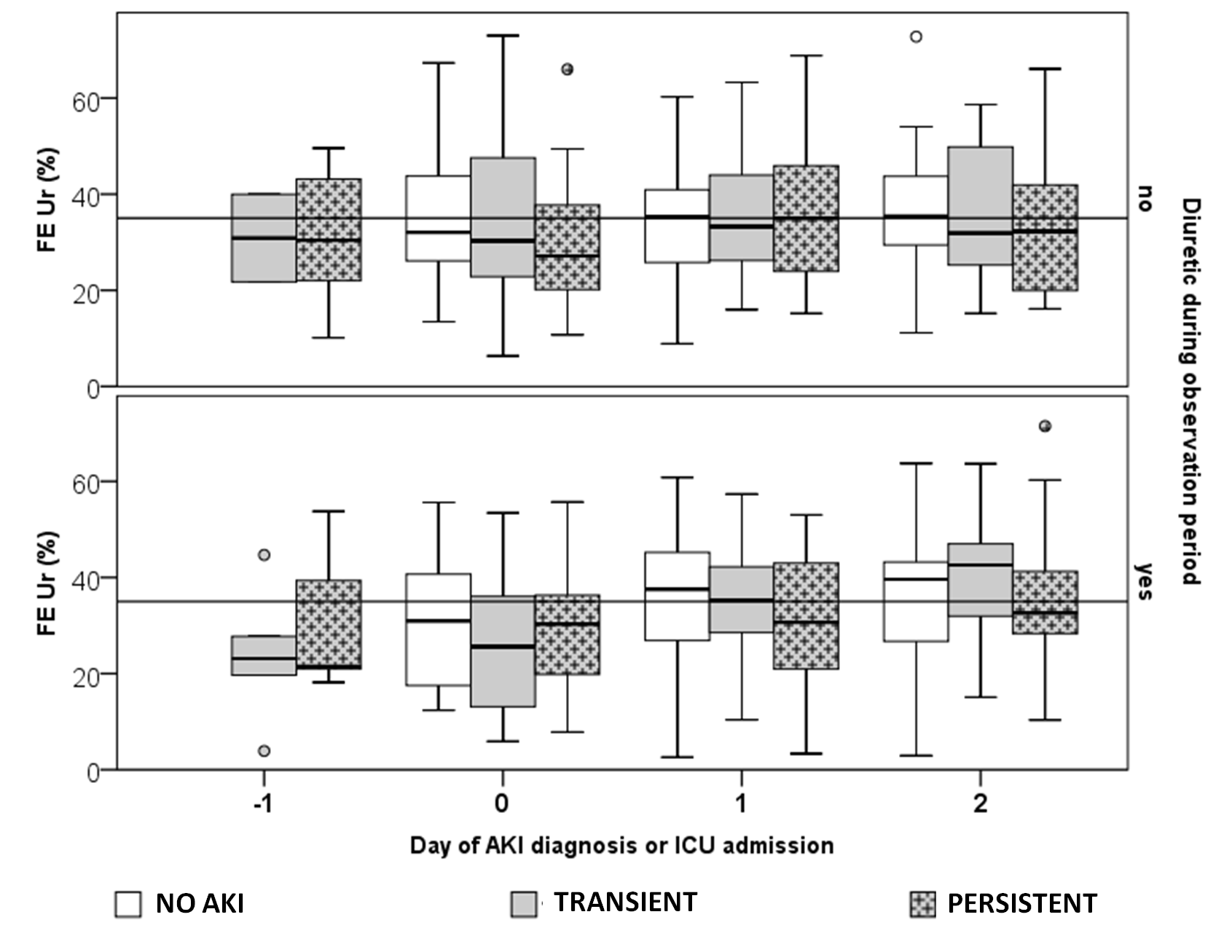

Supplement: Additional file 2: Figure S2 — Fractional excretion of urea (FEUr) between 1 day before (-1) until 2 days after (2) acute kidney injury (AKI) diagnosis (0), according to AKI duration and diuretic use during the observation period. [file 1471-2253-13-31-S2.tiff]
